# Meisoindigo, but not its core chemical structure indirubin, inhibits zebrafish interstitial leukocyte chemotactic migration

**DOI:** 10.1080/13880209.2016.1238949

**Published:** 2016-12-16

**Authors:** Baixin Ye, Xiaoxing Xiong, Xu Deng, Lijuan Gu, Qiongyu Wang, Zhi Zeng, Xiang Gao, Qingping Gao, Yueying Wang

**Affiliations:** a Department of Hematology, Renmin Hospital of Wuhan University, Wuhan, Hubei, China;; b State Key Laboratory of Medical Genomics and Shanghai Institute of Hematology, Rui Jin Hospital, Shanghai Jiao Tong University School of Medicine, Shanghai, China;; c Department of Neurosurgery, Renmin Hospital of Wuhan University, Wuhan, Hubei, China;; d College of Chemistry and Molecular Science, Wuhan University, Wuhan, Hubei, China;; e Central Laboratory, Renmin Hospital of Wuhan University, Wuhan, Hubei, China;; f Department of Pathology, Renmin Hospital of Wuhan University, Wuhan, Hubei, China

**Keywords:** Meisoindigo, indirubin, leukocyte chemotactic migration, zebrafish, inflammation

## Abstract

**Context:** Inflammatory disease is a big threat to human health. Leukocyte chemotactic migration is required for efficient inflammatory response. Inhibition of leukocyte chemotactic migration to the inflammatory site has been shown to provide therapeutic targets for treating inflammatory diseases.

**Objective:** Our study was designed to discover effective and safe compounds that can inhibit leukocyte chemotactic migration, thus providing possible novel therapeutic strategy for treating inflammatory diseases.

**Materials and methods:** In this study, we used transgenic zebrafish model (Tg:*zlyz*-EGFP line) to visualize the process of leukocyte chemotactic migration. Then, we used this model to screen the hit compound and evaluate its biological activity on leukocyte chemotactic migration. Furthermore, western blot analysis was performed to evaluate the effect of the hit compound on the AKT or ERK-mediated pathway, which plays an important role in leukocyte chemotactic migration.

**Results:** In this study, using zebrafish-based chemical screening, we identified that the hit compound meisoindigo (25 μM, 50 μM, 75 μM) can significantly inhibit zebrafish leukocyte chemotactic migration in a dose-dependent manner (*p* = 0.01, *p* = 0.0006, *p* < 0.0001). Also, we found that meisoindigo did not affect the process of leukocyte reverse migration (*p* = 0.43). Furthermore, our results unexpectedly showed that indirubin, the core structure of meisoindigo, had no significant effect on zebrafish leukocyte chemotactic migration (*p* = 0.6001). Additionally, our results revealed that meisoindigo exerts no effect on the Akt or Erk-mediated signalling pathway.

**Discussion and conclusion:** Our results suggest that meisoindigo, but not indirubin, is effective for inhibiting leukocyte chemotactic migration, thus providing a potential therapeutic agent for treating inflammatory diseases.

## Introduction

Inflammation is a protective response to prevent the spread of infection, followed by the process of recovery which is to restore the affected tissues to their normal structural and functional state (Medzhitov 2010). Leukocyte chemotactic migration refers to a multistep process of directed leukocyte migration upon the stimulation of chemotactic factors, which is required for efficient inflammatory response (Friedl & Weigelin 2008). Upon induction of inflammation, leukocytes undergo chemotactic migration and are recruited to the inflammatory sites to eliminate the initial inflammatory causes. Many lines of evidence showed that overreacted leukocytes recruitment in the inflamed region led to some inflammatory diseases such as rheumatoid arthritis, asthma, chronic obstructive pulmonary disease, multiple sclerosis (MS), Crohn’s disease and ulcerative colitis (Friedl & Weigelin 2008; Mackay 2008; Nathan & Ding 2010). It was reported that mutations of genes involved in cell migration system were closely related to the susceptibility to some inflammatory diseases (Mackay 2008). Additionally, PI3K inhibitor was reported to efficiently ameliorate mouse rheumatoid arthritis by inhibiting leukocyte migration (Camps et al. 2005). Thus, given inflammatory diseases are the big threat of human health and discovering anti-inflammatory agents is an urgent need (Dinarello 2010), identification of agents that target leukocyte migration could lead to therapeutic strategies for treating these diseases.

Indirubin, the active ingredient of Chinese traditional medicine Qing Dai, which is one of the herbal medicine of *Danggui Luhui Wan*, was reported to be effective for treating chronic myeloid leukaemia (CML) by inhibiting leukaemia cells proliferation and inducing their apoptosis (Hoessel et al. 1999; Liu et al. 2001; Xiao et al. 2002). However, indirubin exhibited poor water solubility and had severe side effects of gastrointestinal tract in some patients (Cooperative Group of Clinical Therapy of Indirubin 1980; Zheng et al. 1979). To develop a new agent derived from indirubin with high efficiency and low toxicity, a series of derivatives were synthesized and structure-activity relationships were investigated (Ji & Zhang 1985; Wu et al. 1984, 1985). Meisoindigo, also known as Jia Yidian in Chinese, structurally similar to indirubin, was developed with a methyl group at N element (Figure 4(a)). It’s revealed that meisoindigo exhibited higher antitumor activity than indirubin not only in animal models but also in clinical trials (Ji et al. 1985). Meisoindigo was reported as an effective drug in the treatment of CML patients in a Chinese long-term outcome observation (Liu et al. 2001). In maintaining treatment, meisoindigo produced longer chronic phase and survival and lower percentage of transformation in CML patients as compared with those using busulfan, a classic drug for CML treatment. However, with the development and approval of the effective drug imatinib in CML target therapy in 2001, nearly 90% of the initial CML patients treated with imatinib have exhibited no disease progression today (Thompson 2009). Imatinib with a surprising effect provided a better choice for CML treatment, so meisoindigo did not receive further studied in the treatment of CML. Given that meisoindigo is a promising lead compound with clinical safety and antileukemia effect, discovering its novel biological activities is very important for the effective drug development in some diseases other than CML.

Zebrafish, a vertebrate animal model with characteristics of small size, transparent body, and morphological or physiological similarity to mammals, is widely used in the study of immunity and phenotype-driven chemical screening (Zon & Peterson 2005; Lieschke & Currie 2007). The genome of zebrafish is highly conserved with human and mouse, making zebrafish have considerable relevance over other traditional models such as *Drosophila* or *Caenorhabditis elegans*, which lack genes that are involved in some aspects of immunity and other functions (Trede et al. 2004; Yoder et al. 2002), Compared with the mouse model, zebrafish embryo is transparent and thus we can use the fluorescent microscope to observe and analyze the immunological response lively and dynamically (Yaniv et al. 2006). Especially, it has been shown that zebrafish is a desirable tool for the study of leukocyte chemotactic migration *in vivo* (Hall et al. 2007; Mathias et al. 2006; Niethammer et al. 2009; Renshaw et al. 2006; Zhang et al. 2008). The dynamic behaviour of leukocytes in zebrafish embryo upon acute inflammatory stimulation was observed and analyzed under fluorescent microscope at alive and whole animal, which facilitated us to have deep insights into the migrating course of leukocytes in inflammatory response (Niethammer et al. 2009; Tobin David et al. 2012; Yoo et al. 2011). We have previously constructed a transgenic zebrafish line Tg:*zlyz*–enhanced GFP (EGFP) for tracking leukocyte biological behaviours *in vivo* (Zhang et al. 2008). In Tg*:zlyz*-EGFP embryos (about 3 days post-fertilization, dpf), the EGFP positive cells mainly contained the subsets of monocytes/macrophages and neutrophils, both of which are called ‘leukocytes’ and play an important role in acute inflammation (Liu et al. 2013; Ye et al. 2015; Zhang et al. 2008). Given that the body of zebrafish embryo is very small, we used this model (Tg:*zlyz*-EGFP) to screen the hit compounds for inhibiting leukocyte chemotactic migration as previously described (Liu et al. 2013; Ye et al. 2015). Thus, using zebrafish model to screen compounds that can modulate leukocyte migration behaviour is applicable for the discovery and development of anti-inflammatory drugs.

The potential application of meisoindigo in treating inflammatory diseases has not been clearly charted yet (Blazevic et al. 2015). In this study, using transgenic zebrafish model Tg:*zlyz*-EGFP, we identified that meisoindigo can exert inhibitory effect on zebrafish leukocyte chemotactic migration to the injury site without affecting their reverse migration, implying that meisoindigo might be a potential drug lead in the treatment of inflammatory diseases. Interestingly, we also found that indirubin, the core chemical structure of meisoindigo, could not inhibit zebrafish leukocyte migration, suggesting that meisoindigo has unique biological activities and exerts a novel underlying molecular mechanism.

## Materials and methods

### Zebrafish husbandry

The breeding transgenic zebrafish line (Tg:*zlyz*-EGFP) was maintained at 28.5 °C in a circulating water system on a 12 h light/dark cycle. Zebrafish embryos were collected by natural spawning, cultured in egg water (60 μg/mL ‘Instant Ocean^®^’ sea salt in distilled water) and were staged according to Kimmel et al. (1995). The leukocyte-specific zebrafish line Tg:*zlyz*-EGFP was used for all drug screening and inflammation assays at 3 dpf as previously described (Liu et al. 2013; Zhang et al. 2008). All animal experiments have been approved by Department of Animal Experimentation at Shanghai Jiao Tong University School of Medicine.

### Reagents and drug treatment

We screened four drugs including arsenic trioxide, meisoindigo, indirubin and tanshinone IIA. Meisoindigo was purchased from Shanghai Yansheng industrial (Shanghai, China). Indirubin, tanshinone IIA and arsenic trioxide were obtained from Shanghai Institute of Hematology, RuiJin Hospital, Shanghai Jiao Tong University School of Medicine, Shanghai, 200025, China (Wang et al. 2008). All compounds were dissolved in DMSO. Then, all compounds were diluted in the egg water containing 0.5% DMSO, and egg water with 0.5% DMSO was applied as a negative control. The volume of drug solution was the same in all assays.

### Leukocyte chemotactic migration in chemical screening

**Figure 1.**
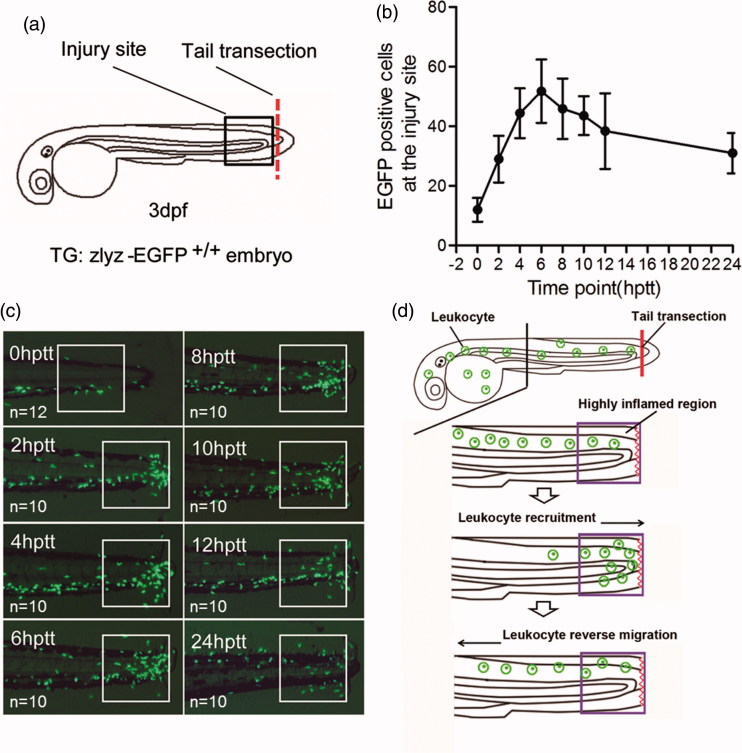
Leukocyte migration in response to acute injury is visualized in zebrafish embryo model (Tg:zlyz-EGFP). (a).Tg:zlyz-EGPF embryos (at 3dpf) were subjected with tail transection for generating zebrafish leukocyte migration model. The red line means the incision of tail transection, and the square region means the highly inflamed region. (b–c). Leukocytes that migrated to the highly inflamed regions [white boxes in (b)] in Tg:zlyz-EGFP embryos (3 dpf, *n* denotes the number of zebrafish embryos) with tail transection were quantitatively analyzed at different time points(c). (d). Schematic diagram of zebrafish leukocyte recruitment and resolution in acute inflammation.

Using transgenic zebrafish (Tg:*zlyz*-EGFP) embryo as an *in vivo* model, we studied the effect of screening compounds on leukocyte recruitment during the induction phase of inflammation as previously reported (Liu et al. 2013; Ye et al. 2015; Zhang et al. 2008). All zebrafish embryos were subjected with tail transection. As shown in Figure 1(a), to induce acute inflammation, the tail of transgenic zebrafish (Tg:*zlyz*-EGFP) embryo (at 3dpf) was subjected to transverse transection by sterile scalpel without impairing the tail circulation. To analyze the leukocyte migration, we used fluorescent microscope to lively observe and photograph the EGFP positive cells in the zebrafish embryo at different time points. From 0 hptt (h post tail transection) to 6 hptt, zebrafish leukocyte takes chemotactic migration to the injury site during the induction phase of inflammation, thus we used this course of leukocyte migration to screen the hit compounds. In chemical screening, the embryos were exposed to the investigated compounds after tail transection. The zebrafish embryos (at 3 dpf, 6 embryos per well) with tail transection were incubated with screening chemicals (25 μM) for 6 h in 96-well plate. At 6 hptt, we arranged the tested embryos in a line under the fluorescent microscope and then photographed the leukocytes (EGFP positive cells) distributed around the body of embryos; In the photograph, the EGFP positive cells (green points) in the highly inflamed region around the injury tail defined by a white box (as shown in Figure 1(a,c)) were counted; By analyzing the number of leukocytes around the highly inflamed injury site at 6 hptt, we can identify the hit compounds that can inhibit zebrafish leukocyte chemotactic migration.

### Leukocyte reverse migration assay in zebrafish model

According to previous reports (Mathias et al. 2006; Robertson et al. 2014), in order to terminate the inflammation, zebrafish leukocytes undergo apoptosis or leave away from the injury site during the resolution phase of inflammation. To test the effect of the screened compounds on leukocyte reverse migration, we incubated tail-transected zebrafish embryo with screening compounds from 6 hptt (a time point at which leukocyte number increased to the highest level) to 12 hptt (a time point at which the compounds accelerating leukocyte resolution can be easily identified), and then analyzed the zebrafish leukocyte number in the highly inflamed region at 12 hptt.

### Western blot analysis

Zebrafish embryos were treated by meisoindigo or indirubin. Extraction of whole zebrafish embryo protein was described in our previous work (Ye et al. 2015; Zhang et al. 2008). The proteins separated by SDS-PAGE electrophoresis were transferred to ECL nitrocellulose membranes and incubated with the primary antibody overnight at 4 °C, followed by incubation of HRP-linked secondary antibody (Cell Signalling Technology) for 1 h at room temperature. According to the manufacturer’s instructions of the equipment (LAS 4000, Fujifilm), detection was performed by an Immobilon Western chemiluminescent HRP substrate kit (Millipore, catalog no. WBKLS0100). The primary antibodies in this study are specific for p-AKT, AKT, p-ERK and ERK (Cell signalling Technology).

### Statistical analysis

Data were presented as mean values ± SD. Statistical analysis was performed using the Graphpad Prism (La Jolla, CA) software. The probability level for statistical significance was *p* < 0.05. 

## Results

### Leukocyte chemotactic migration in response to acute injury can be visualized in transgenic zebrafish model Tg:*zlyz*-EGFP

As reported in our previous work (Liu et al. 2013; Ye et al. 2015; Zhang et al. 2008), the leukocyte migration in response to acute tail injury can be visualized under the fluorescent microscope, and the subsets of EGFP positive cells in transgenic zebrafish line Tg:*zlyz*-EGFP represented the cell group of leukocytes (Figure 1(a)). To show the whole process of leukocyte recruitment and reverse migration in response to acute injury, we recorded the pictures of leukocytes located in the highly inflamed region around zebrafish embryo tail at different time points during 24 h post transection. As shown in Figure 1(b,c), from 0 hptt to 6 hptt, the number of the EGFP positive cells in the highly inflamed region was increased, suggesting that leukocytes were migrating into the injury site during initial phase of inflammation. Then, after the time point of 6 hptt, the number of EGFP positive cells in the highly inflamed region was decreased, suggesting leukocytes retreated from the injury site at the resolution phase of inflammation. In this assay, we found that 6 hptt was a suitable time point to distinguish the recruitment phase from resolution phase of acute inflammatory response. In addition, we concluded that this transgenic zebrafish line Tg:z*lyz*-EGFP is a desirable animal model to visualize the entire process of leukocyte recruitment and reverse migration in acute inflammation during a short time (Figure 1(d)).

### Identification of meisoindigo that inhibits zebrafish leukocyte chemotactic migration to the injury site

**Figure 2.**
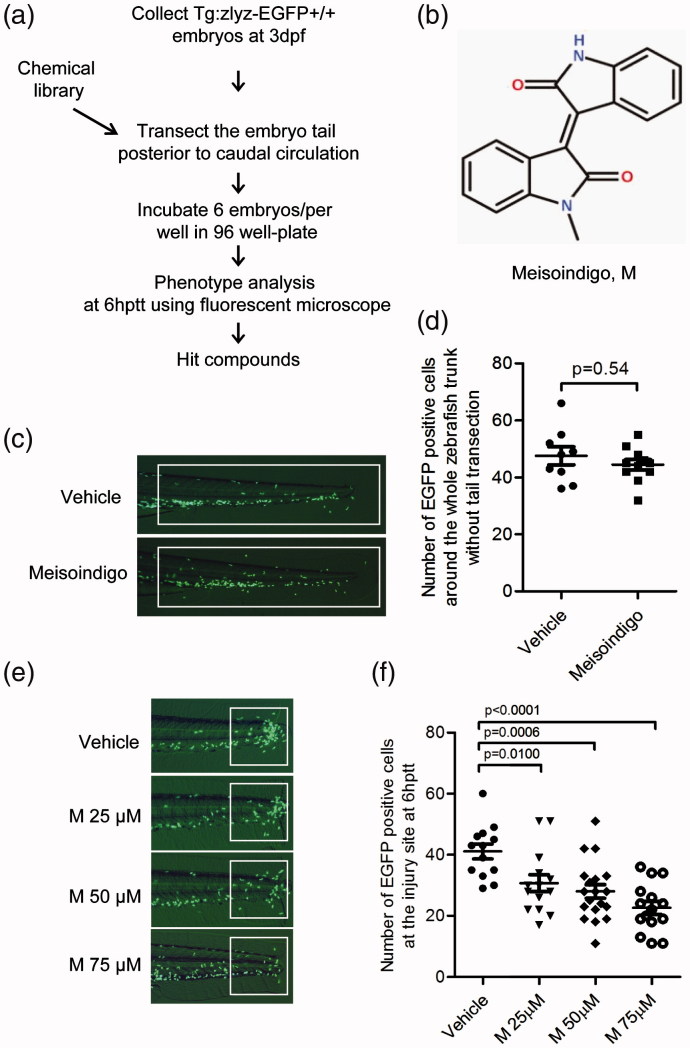
Identification of meisoindigo that inhibits zebrafish leukocyte recruitment to the injury site. (a) Chemical screening strategy. (b) Chemical structure of meisoindigo. (c,d) Tg:zlyz-EGFP embryos (3 dpf) without tail transection were treated with vehicle(*n* = 9) or meisoindigo(M, 100 μM, *n* = 11) for 6 h. Leukocytes around the whole trunk of zebrafish embryo [white boxes in (c)] were quantitiatively analyzed. (e,f) Tg:zlyz-EGFP embryos (3 dpf) with tail transection were treated with vehicle (*n* = 13), 25 μM (*n* = 13), 50 μM (*n* = 18) or 75 μM (*n* = 14) meisoindigo, respectively. Leukocytes that migrated to the highly inflamed regions [white boxes in (e)] at 6 hptt were quantitatively analyzed (f). The *p* values were annotated as obtained from a two-tailed *t* test.

To identify effective compounds that could regulate leukocyte recruitment, we performed a chemical screening using this transgenic zebrafish model Tg:*zlyz*-EGFP (Liu et al. 2013; Ye et al. 2015). The zebrafish embryos at 3 dpf were subjected with tail transection and treated with compounds including arsenic trioxide, meisoindigo, indirubin and tanshinone IIA from 0 hptt to 6 hptt. The effects of these compounds (at 25 μM) on leukocyte recruitment were evaluated by counting the number of EGFP positive cells in the highly inflamed region (Figure 2(a)). Our screening results showed that meisoindigo was a hit compound that could exert inhibitory effect on zebrafish leukocyte chemotactic migration (Table 1). It was found that meisoindigo (Figure 2(b)) does not significantly affect the total number of EGFP positive cells around the whole trunk of zebrafish (*p* = 0.54) (Figure 2(c,d)), suggesting that the apoptosis or proliferation of zebrafish leukocytes might not be regulated upon the treatment of meisoindigo (100 μM). By comparison, the number of EGFP positive cells in the highly inflamed region was decreased significantly in a dose-dependent manner upon the treatment of meisoindigo on zebrafish embryo with tail transection (Figure 2(e,f)). As shown in Figure 2(e), the number of leukocytes in the white-box caged area was lowered by the treatment of meisoindigo at the concentration of 25 μM (*p* = 0.01, *n* = 13), 50 μM (*p* = 0.0006, *n* = 18) or 75 μM (*p* < 0.0001, *n* = 14), while the number of leukocytes around the trunk of zebrafish embryo was increased accordingly. Given the previous observation showing that meisoindigo might not regulate the total number of leukocytes around the whole trunk, decreased migration of leukocytes to the injury site contributed to the decreased number of leukocytes in the inflamed region. Thus, our results indicated that meisoindigo inhibited zebrafish leukocyte chemotactic migration to the injury site.

**Table 1.** List of the compounds in the chemical screening.

Compound	Concentration	Biological effectof inhibiting leukocytechemotatic migration
Arsenic trioxide	25 μM	No effect
Meisoindigo	25 μM	Significantly effective
Indirubin	25 μM	No effect
Tanshinone II A	25 μM	No effect

### Meisoindigo does not affect zebrafish leukocyte reverse migration from the injury site

**Figure 3.**
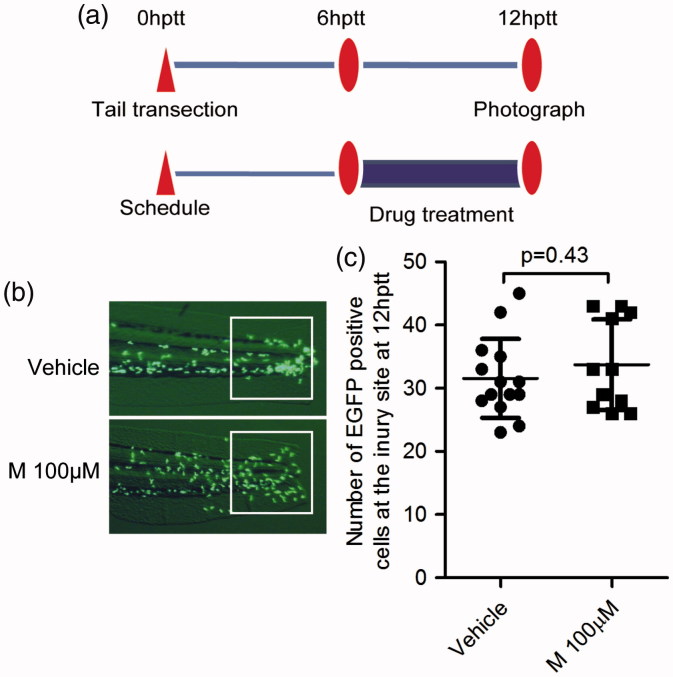
Meisoindigo has no effect on zebrafish leukocyte reverse migration from the injury site. (a) Treatment schedule. Tg:zlyz-EGFP embryos (3dpf) were subjected with tail transection and administrated with vehicle (*n* = 14) or meisoindigo (M, 100 μM, *n* = 11) from 6 hptt to 12 hptt. (b, c) Leukocytes migrated to the highly inflamed regions [white boxes in (b)] at 12hptt were quantitatively analyzed (c). The *p* value was annotated as obtained from a two-tailed *t* test.

The leukocyte chemotactic migration to the injury site is usually followed by leukocyte reverse migration from the highly inflamed region. This process plays an important role at the resolution phase of inflammation (Nathan & Ding 2010). The regulated pathways of leukocyte chemotactic migration are different from those in regulating leukocyte reverse migration (Robertson et al. 2014). To figure out whether meisoindigo can regulate the process of leukocyte reverse migration, we treated the tail-transected zebrafish embryo (3 dpf) with meisoindigo from 6 hptt to 12 hptt (Figure 3a). At 12 hptt, the number of EGFP positive cells in the highly inflamed region of zebrafish embryos was not significantly different between the control and the group treated with meisoindigo (Figure 3(b,c), *p* = 0.43). This result showed that meisoindigo has no effect on the reverse migration of zebrafish leukocytes.

### Indirubin could not inhibit leukocyte chemotactic migration

**Figure 4.**
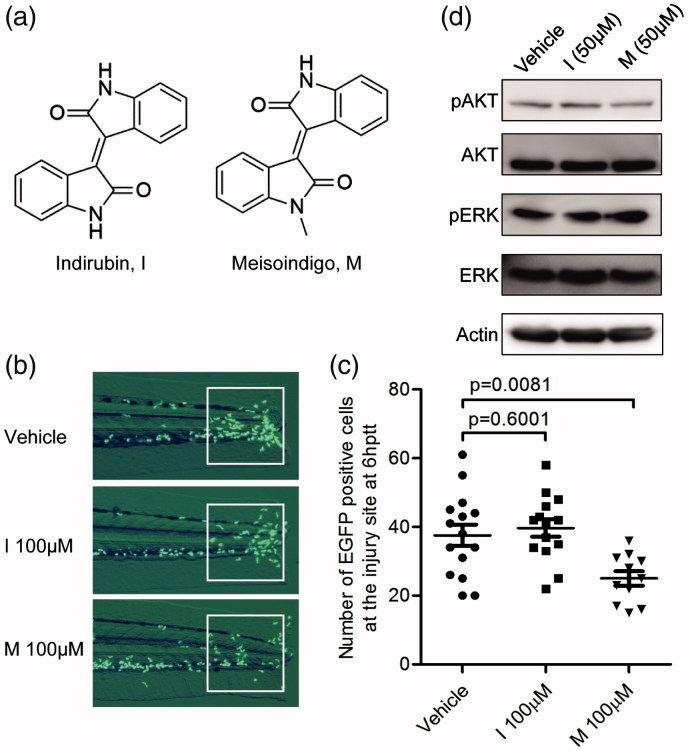
Indirubin, the core chemical structure of meisoindigo, did not inhibit leukocyte chemotactic migration, and meisoindigo does not regulate pAKT/AKT or pERK/ERK level. (a) Chemical structure of indirubin (I) and meisoindigo (M). Meisoindigo is a synthetic derivative of indirubin with a methyl substitution at N element. (b,c) Tg:zlyz-EGFP embryos (3dpf) with tail transection were treated with vehicle (*n* = 15), indirubin (I, 100 μM, *n* = 14) or meisoindigo (M, 100 μM, *n* = 11), respectively. Leukocytes that migrated to the highly inflamed regions [white boxes in (b)] at 6 hptt were quantitatively analyzed (c). The *p* values were annotated as obtained from a two-tailed *t* test. (d) Westernblot analysis showing the effect of meisoindigo on the selected signaling pathway components. 20 zebrafish embryos (3dpf, *n* = 20) with tail transection were treated with vehicle, meisoindigo or indirubin at the indicated concentration from 0 hppt to 6 hptt, respectively. Then, the lysate of whole zebrafish embryos was subjected to westernblot analysis for detecting pAKT/AKT or pERK/ERK level.

Indirubin, the Chinese traditional medicine, is the core chemical structure of meisoindigo (Figure 4(a)). Both indirubin and its derivative meisoindigo have been used in the treatment of chronic myeloid leukemia (CML) (Blazevic et al. 2015). By comparison, meisoindigo was reported to be more effective and soluble than indirubin (Hoessel et al. 1999). To test the activity of indirubin on the zebrafish leukocyte chemotactic migration, we treated tail-transected zebrafish embryos with meisoindigo or indirubin and compared the number of leukocytes around the tail of zebrafish embryos. As shown in Figure 4(b,c), we found that indirubin treatment (100 μM) did not influence the number of EGFP positive cells in the highly inflamed region (*p* = 0.6001), while meisoindigo treatment significantly led to an inhibitory effect. This observation suggested that meisoindigo, but not its core chemical indirubin, can inhibit leukocyte chemotactic migration in the zebrafish embryo.

### Meisoindigo does not regulate the phosphorylated AKT or ERK level

Next, we wanted to make clear which signalling pathways are responsible for meisoindigo’s inhibitory effect on leukocyte chemotactic migration. Given that AKT or ERK mediated pathways play an important role in this process (Ren et al. 2015; Sapharikas et al. 2015; Yoo et al. 2010), we analyzed the alterations of selected pathways including the AKT or ERK involved pathways in zebrafish embryos upon meisoindigo treatment. Unexpectedly, western blot analysis showed that the levels of phosphorylated AKT and ERK were not markedly changed (Figure 4(d)). This result suggested that meisoindigo might regulate leukocyte chemotactic migration in an AKT or ERK-independent manner, and might perform its biological function by other alternative mechanism.

## Discussion

Meisoindigo, a chemical derived from the Chinese traditional medicine indirubin, has been used in the treatment of CML without inducing uncontrolled toxicity, suggesting meisoindigo might be a promising drug that can be well tolerated for the patients (Liu et al. 2001). In this study, we firstly investigated the biological activity of meisoindigo on leukocyte chemotactic migration in transgenic zebrafish model, and found that it posed an inhibitory effect on leukocyte chemotactic migration without affecting its reverse migration. Interestingly, our results also showed that indirubin, the core chemical structure of meisoindigo, did not show similar effect of inhibiting leukocyte chemotactic migration. In addition, we found that meisoindigo did not regulate the level of phosphorylated Akt and ERK, which was considered to be involved in leukocyte chemotactic migration. These results suggested that meisoindigo might have potential clinic implication in the treatment of inflammatory diseases and function by alternative underlying mechanisms to regulate leukocyte chemotactic migration.

Zebrafish model is a desirable model organism that could be used to discover novel compounds for treating inflammatory diseases (Lieschke & Currie 2007; Zon & Peterson 2005). We previously used the transgenic zebrafish line Tg:*zlyz*–EGFP to discover a series of compounds that can regulate leukocyte recruitment to the highly inflamed region (Liu et al. 2013; Ye et al. 2015). Especially, we identified that vibsanin B, a novel natural product isolated from *Viburnum odoratissimum Ker-Gawl*, can inhibit zebrafish leukocyte recruitment to the highly inflamed region and ameliorate mice experimental autoimmune encephalomyelitis, implying that zebrafish can be used in the discovery of novel anti-inflammatory agents (Ye et al. 2015). According to our previous report, our screened natural product vibsanin B that inhibits zebrafish leukocyte chemotactic migration was effective for the treatment of murine experimental autoimmune encephalomyelitis (EAE) model (Ye et al. 2015). Similarly, in this study we used zebrafish model to visualize the whole process of leukocyte chemotactic migration and reverse migration (Figure 1). Furthermore, we identified meisoindigo as a safe, effective and well-tolerated drug in treating CML patients can inhibit zebrafish leukocyte chemotactic migration in a dose-dependent manner at the whole animal level (shown in Figure 2). This primary observation implies that meisoindigo as an effective drug for treating CML might be newly used in treating some inflammatory diseases. Interestingly, it was reported that meisoindigo was used to ameliorate psoriasis (Zhang et al. 2013), an autoimmune inflammatory disease, suggesting that meisoindigo is a potential hit compound that can be applied in the treatment of some inflammatory diseases. Given that zebrafish model in the study of inflammation could not replace the mouse and human cell model, using human monocyte/macrophage or neutrophil cell lines (THP-1 cells or HL60 cells) in transwell system or mouse inflammatory diseases model to evaluate the activity of meisoindigo is desirable for the further study as previously reported (Liu et al. 2013; Ye et al. 2015). Thus, preclinical or clinical studies for the application of meisoindigo in treating some inflammatory diseases are necessary in the future drug development.

Meisoindigo with a methyl substitution at N element was very similar with indirubin in chemical structure and biological activity in treating CML patients in China. As shown in Figure 4, meisoindigo was shown to exert inhibitory effect on zebrafish leukocyte chemotactic migration. In contrast, indirubin does not show the inhibitory effect on leukocyte chemotactic migration. This phenotypic difference suggested that meisoindigo might bind its unique functional target to regulate zebrafish leukocyte chemotactic migration, while indirubin might not bind this functional target and thus lead to a negative result. According to the previous reports, indirubin can target the ATPase binding site of cyclin-dependent kinases (CDKs) and potentially inhibit their biological activity, which contributes to the application of indirubin in cancer treatment (Hoessel et al. 1999). Unexpectedly, meisoidigo as the derivative of indirubin could not affect the activity of CDKs, and thus the direct target of meisoindigo might not be the same as its core chemical structure indirubin. (Wee et al. 2009). This conclusion was consistent with our primary observation that meisoindigo produced different phenotype from indirubin in leukocyte chemotactic migration. Thus, identifying the direct target of meisoindigo is very important for understanding its mechanism of action and facilitating its clinic application in the future.

Many lines of evidence indicated that PI3K-AKT pathway components can regulate the moving directionality of leukocytes and play a key role in leukocyte chemotactic migration (Yoo et al. 2010; Zhang et al. 2008). In zebrafish embryos, the PI3Kγ-AKT pathway promotes Rac mediated actin polymerization around the leading edge, which guides leukocyte chemotactic migration *in vivo* (Yoo et al. 2010). In our previous report, we also found that the natural product vibsanin B can markedly decreased the phosphorylated AKT and thus led to the impairment of leukocyte chemotactic migration into the injury site (Ye et al. 2015). However, as shown in Figure 4(d), we interestingly found that the screened compound meisoindigo did not affect the phosphorylated AKT level, suggesting that meisoindigo might regulate zebrafish leukocyte migration in an AKT-independent manner. This effect of meisoindigo on AKT mediated pathway was inconsistent with the classic mechanism by some existing compounds such as vibsanin B (Ye et al. 2015). In some cases, the regulation of meisoindigo on leukocyte chemotactic migration was not realized by the classic PI3K-AKT pathway (Volpe et al. 2010). Similarly, previous reports showed that the ERK-mediated pathway was also involved in the process of leukocyte chemotactic migration (Ren et al. 2015; Sapharikas et al. 2015), while in our system we could not detect the regulation of phosphorylated ERK level (Figure 4(d)). Thus, meisoindigo might take some other alternative underlying mechanisms to inhibit leukocyte chemotactic migration. For example, it was recently reported that leukocyte function can be modulated by plant- and bacteria-derived compounds through regulating intracellular calcium concentration in leukocytes (Nowak et al. 2015). It is very interesting to figure out whether meisoindigo could influence the intracellular calcium concentration to modulate leukocyte chemotactic migration. Thus, understanding the molecular mechanism of meisoindigo in leukocyte chemotactic migration needs further investigation in the future study.
